# Delineating taxonomic boundaries in the largest species complex of black flies (Simuliidae) in the Oriental Region

**DOI:** 10.1038/srep20346

**Published:** 2016-02-03

**Authors:** Van Lun Low, Hiroyuki Takaoka, Pairot Pramual, Peter H. Adler, Zubaidah Ya’cob, Yao-Te Huang, Xuan Da Pham, Rosli Ramli, Chee Dhang Chen, Anukhcha Wannaket, Mohd Sofian-Azirun

**Affiliations:** 1 Institute of Biological Sciences, Faculty of Science, University of Malaya, Kuala Lumpur, Malaysia; 2 Department of Biology, Faculty of Science, Mahasarakham University, Maha Sarakham, Thailand; 3 Department of Agricultural and Environmental Sciences, Clemson University, Clemson, SC, USA; 4 Fuji Environmental Service, Mitsuwa, Kawaguchi City, Saitama, Japan; 5 National Institute of Food Control, Ministry of Health, Hanoi, Vietnam

## Abstract

Perspicuous assessments of taxonomic boundaries and discovery of cryptic taxa are of paramount importance in interpreting ecological and evolutionary phenomena among black flies (Simuliidae) and combating associated vector-borne diseases. *Simulium tani* Takaoka & Davies is the largest and perhaps the most taxonomically challenging species complex of black flies in the Oriental Region. We use a DNA sequence-based method to delineate currently recognized chromosomal and morphological taxa in the *S. tani* complex on the Southeast Asian mainland and Taiwan, while elucidating their phylogenetic relationships. A molecular approach using multiple genes, coupled with morphological and chromosomal data, supported recognition of cytoform K and morphoform ‘b’ as valid species; indicated that *S. xuandei*, cytoform L, and morphoform ‘a’ contain possible cryptic species; and suggested that cytoform B is in the early stages of reproductive isolation whereas lineage sorting is incomplete in cytoforms A, C, and G.

Genetic variation and differentiation have long been of interest to biologists^1^,^2^, and recognition of multiple lineages within taxa is a primary step in understanding evolutionary processes. Nonetheless, recognition of these lineages can be problematic when genetic differentiation within morphologically conserved taxa is minimal^3^. With the advent of molecular techniques, a DNA sequence-based approach has proven particularly powerful for identifying lineages, discovering cryptic diversity, and delimiting species boundaries^4^,^5^,^6^.

The *Simulium tuberosum* species group is one of the most structurally uniform taxa of black flies, with species typically differing only in subtle characteristics of one or two life stages, such as pupal gill configuration^7^. The group is well known for its species richness and abundance across the Holarctic and Oriental regions^7^,^8^. The most diverse taxon in the *S. tuberosum* group is the *S. tani* Takaoka & Davies species complex, with nine known cytoforms (A–I) in Thailand, one cytoform (K) in Malaysia, one cytoform (L) in Taiwan^9^,^10^,^11^, and two morphoforms (‘a’ and ‘b’) and one morphospecies (*S. xuandei* Takaoka & Pham) in Vietnam^12^. Diagnostic morphological characters have been found for cytoform H^7^; *S. xuandei*, morphoforms ‘a’ and ‘b’^12^; and cytoforms K and L (unpublished). In addition to the 11 cytoforms previously confirmed chromosomally^9^,^10^,^11^, we have found that morphoforms ‘a’ and ‘b’ and *S. xuandei* are each chromosomally distinct from all other members of the complex, and that ‘a’ consists of two cytoforms (unpublished).

A DNA sequence-based approach using multiple genes provides a robust tool for discovering and delineating biodiversity^13^,^14^,^15^,^16^,^17^. The members of the *S. tani* complex in Thailand and Malaysia have been subjected to several phylogenetic and phylogeographic studies using the COI, COII, and 18S rRNA/ITS1 genes^18^,^19^,^20^. These studies have yet to resolve the taxonomic status of the cytologically distinct members of the complex. However, a phylogeographic analysis based on COI sequences was able to distinguish two forms in Thailand^18^, suggesting some threshold of genetic distinctiveness.

Our primary aim was to elucidate the phylogenetic relationships and taxon boundaries of the members of the *S. tani* complex from the mainland of Southeast Asia and Taiwan (Table 1). We genetically characterized these members, using the mitochondrial COI and COII genes and the fast-evolving nuclear ECP1 gene. We also determined whether the COI and COII genes alone, or in combination with the ECP1 gene, can improve phylogenetic resolution.

## Results

### Genetic distances

The ranges of intraspecific divergence were 0.00–2.41% (cytoforms A and G) for COI, 0.00–1.66% (cytoform G) for COII, and 0.00–2.59% (morphoform ‘a’) for ECP1 (Supplementary Tables S1-S3). Interspecific differentiation among members, using single-locus data was ambiguous, notably for members on the Southeast Asian mainland.

Intraspecific divergence for the COI + COII + ECP1 dataset varied from 0.00 (cytoform C and morphoform ‘b’) to 1.72% (cytoform A). Interspecific differentiation among cytoforms A, B, C and G in Thailand was ambiguous. The highest interspecific divergence (6.79%) was between cytoforms B and L (Table 2).

### Phylogenetic analyses

The mitochondrial COI and COII genes each revealed three monophyletic lineages, corresponding geographically to Thailand-Malaysia, Vietnam, and Taiwan. Within the Thailand-Malaysia lineage, cytoform K formed a sublineage with high bootstrap support. Neither gene unequivocally resolved phylogenetic relationships for the cytologically defined Thai members or the morphologically defined Vietnamese members (Supplementary Figs S1 and S2).

**Figure 1:**
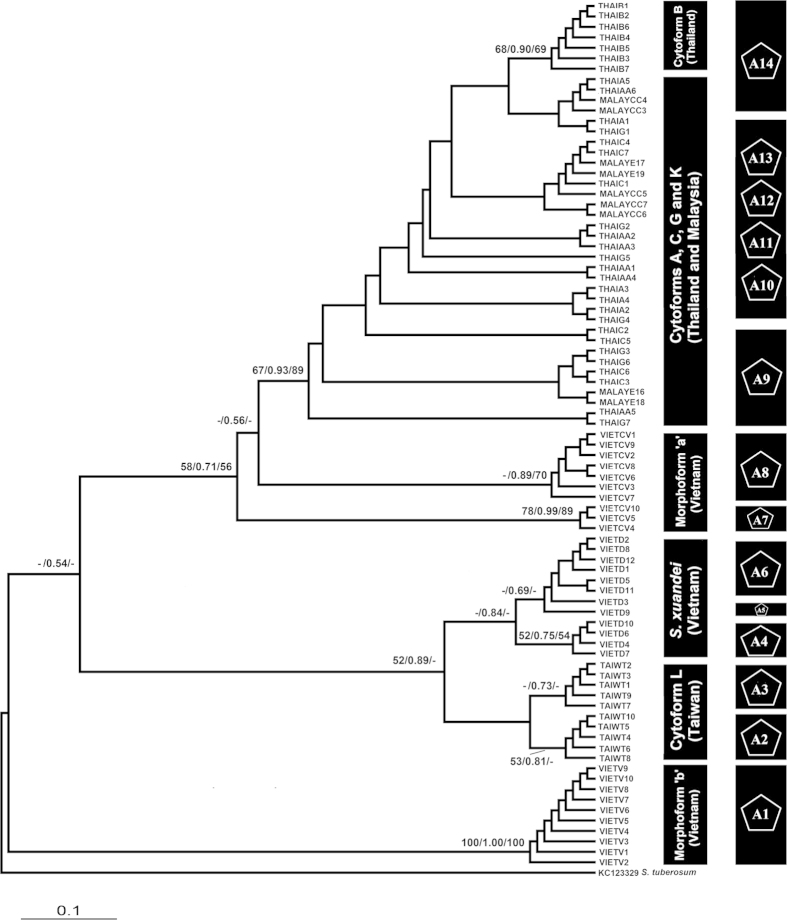
Phylogenetic tree of *Simulium* taxa based on ECP1 sequences. Posterior probability/bootstrap [Maximum likelihood (ML)/Bayesian inference (BI)/neighbour-joining (NJ)] values are shown on the branches. Values less than 0.5/50 are not shown. The scale bar represents 0.1 substitutions per nucleotide position. The second column shows 14 entities/species identified by the GMYC likelihood analysis.

The ECP1 gene distinguished Vietnamese morphoforms ‘a’ and ‘b’ and *S. xuandei*, but did not resolve their relationships (no bootstrap support in the deeper nodes); hence, the nuclear phylogeny was compatible with the mitochondrial topologies in which morphoforms ‘a’ and ‘b’ and *S. xuandei* formed one clade. Nevertheless, morphoform ‘a’, as well as cytoforms A, C, G, and K, were not monophyletic. Cytoforms B and L formed their respective clades. The nuclear phylogeny was consistent with the morphological groupings, whereby morphoform ‘a’ showed a closer relationship with cytoform K, and *S. xuandei* showed a sister relationship with cytoform L. Morphoform ‘b’ represented a distinct genetic lineage (Fig. 1), in agreement with its chromosomal and morphological separation from cytoform K and morphoform ‘a’.

**Figure 2:**
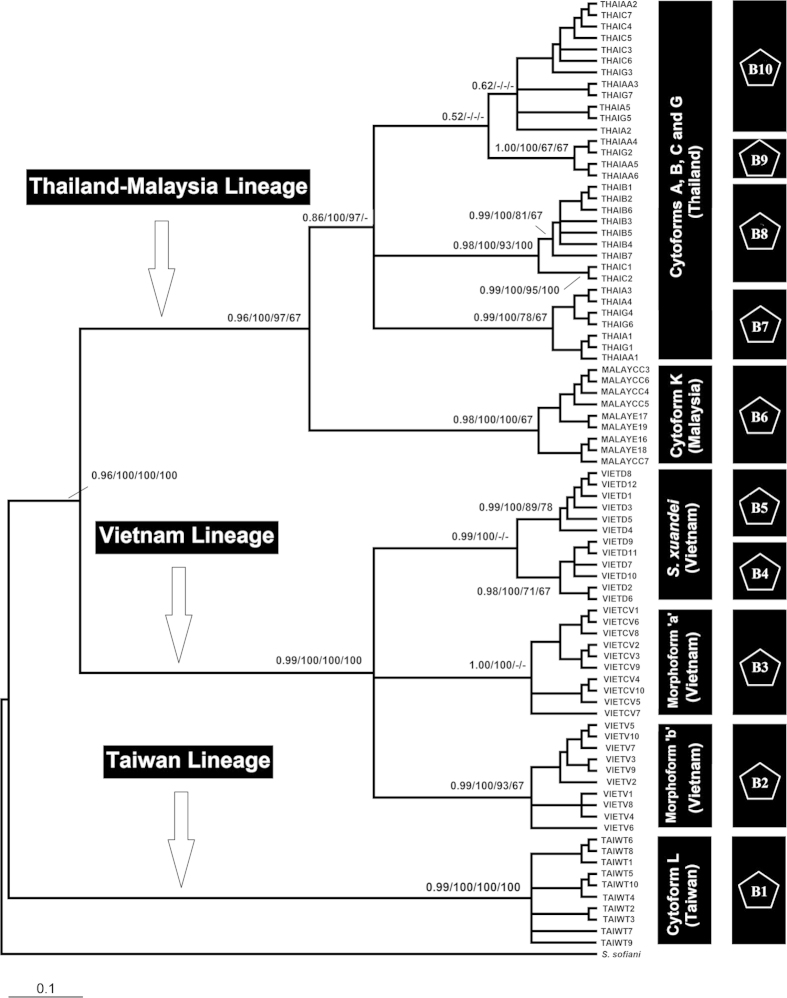
Phylogenetic tree of *Simulium* taxa based on concatenated COI, COII, and ECP1 sequences. Posterior probability/bootstrap [Bayesian inference (BI)/maximum likelihood (ML)/neighbour-joining (NJ)/maximum parsimony (MP)] values are shown on the branches. Values less than 0.5/50 are not shown. The scale bar represents 0.1 substitutions per nucleotide position. The second column shows 10 entities/species identified by the GMYC likelihood analysis.

The concatenated mitochondrial and nuclear dataset provided strong support for three geographically based lineages corresponding to populations in Thailand-Malaysia, Vietnam, and Taiwan. With the exception of the four cytoforms in Thailand, all members were assigned to their respective clades with high bootstrap support (Fig. 2).

### Coalescent delimitation

The nuclear gene GMYC analysis with single threshold model (likelihood of null model: 27.48, ML of GMYC model: 30.40, likelihood ratio: 5.83, LR test: 0.05) revealed 13 ML clusters (confidence interval: 3–18) and 14 entities/species (confidence interval: 3–22). For the concatenated dataset, a total of 10 ML clusters (confidence interval: 2–20) and 10 entities/species (confidence interval: 2–18) were identified (likelihood of null model: 56.99, ML of GMYC model: 61.38, likelihood ratio: 8.77, LR test: 0.01). Neither the concatenated dataset nor the nuclear dataset supported the GMYC entities for cytoforms A, B, C, and G as valid species; the estimated lineages did not correspond with the respective cytoforms. Our results suggested the presence of one or two possible cryptic species in *S. xuandei*, one in morphoform ‘a’ and perhaps one in cytoform L (Figs 1 and 2). Cytoform K and morphoform ‘b’ could be recognized confidently as valid species (Figs 1 and 2).

## Discussion

Morphotaxonomy and cytotaxonomy are common approaches in the classification and species assessments of black flies. These approaches, however, have limitations when taxa are isomorphic or defined only by sex-linked (i.e., non-fixed) rearrangements, as with some members of the *S. tani* complex^11^. DNA barcoding with COI is an alternative approach for species identification and discovery of cryptic species^21^,^22^. Yet, it, too, has limited utility for some morphologically and chromosomally similar species^15^,^16^,^23^,^24^. For instance, it does not resolve the taxonomic status among chromosomally distinct members of the *S. tani* complex in Thailand^20^ or the morphologically defined members in Vietnam. We found that COI sequences are identical among some of these taxa and that intraspecific variation far exceeds the interspecific divergence; hence, species identification using this gene can be equivocal^23^,^25^.

Given that a single locus for delimiting species boundaries can provide limited resolution, we applied a multi-gene approach, recognized for resolving evolutionary relationships in the Simuliidae^15^,^16^. Our results show that the mitochondria-encoded COI, COII, 12S rRNA, and 16S rRNA genes and the hypervariable region of the nuclear-encoded 28S rRNA fail to distinguish our taxa from Vietnam and Thailand, highlighting the need for additional markers. A fast-evolving nuclear gene, ECP1, has been proposed as the gold standard for identification of members of the *S. jenningsi* species group^26^, a congener of the *S. tani* complex. The high performance of this marker prompted its use in our study, and certainly this gene serves our purpose. For example, the Vietnamese taxa and Thai cytoform B could be distinguished. The ECP1 phylogeny also supports the morphological hypotheses of relationships^12^, with morphoform ‘a’ showing a close relationship with cytoform K, and *S. xuandei* showing a sister relationship with cytoform L. Morphoform ‘b’ represents a distinct genetic lineage, in agreement with its morphological distinction—lack of terminal pupal hooks^12^—from cytoform K and morphoform ‘a’. The concatenated dataset improves resolution of the relationships among members of the *S. tani* complex by recovering clades for cytoforms K and L, morphoforms ‘a’ and ‘b’, and morphospecies *S. xuandei*. This dataset also provides strong support for three geographically based lineages corresponding to populations in Thailand-Malaysia, Vietnam, and Taiwan.

Cytoforms A, C, and G are chromosomally similar, differing from one another principally in the role of inversion IIIL-6—fixed, X linked, or Y linked, respectively^11^. Our molecular analyses do not resolve these three cytoforms, a common finding among closely related black flies, particularly those in species complexes^13^,^20^. The lack of monophyly for members of simuliid species complexes often is attributed to introgressive hybridization, inadequate genetic information, and incomplete lineage sorting^5^,^20^,^23^. In our study, the Thai samples were collected from geographically distant populations; hence, mitochondrial introgression between the Thai cytoforms is less likely. The concatenated dataset and nuclear gene demonstrate divergent lineages for other members of the complex; however, we cannot confidently rule out inadequate genetic information as the cause of non-monophyly for the Thai members because weak support was detected for some of the nodes in the phylogenetic trees. Evidence of recent Pleistocene expansion^18^ is in line with rapid and recent lineage radiations of the Thai members; hence, incomplete lineage sorting might be expected. The genetic distances and haplotype relationships between these rapidly radiating taxa support our hypothesis of incomplete lineage sorting, whereby identical sequences are observed in the mitochondrial dataset. We tentatively regard cytoforms A, C, and G as members of a single chromosomally polymorphic species exhibiting the earliest stages of differentiation.

Among the Thai members of the complex, cytoform B is the most distinct, lacking two chromosome inversions (IIIL-5 and IIIL-6) that are shared by cytoforms A, C, and G^11^. The fast-evolving ECP1 gene supports its distinctiveness by recovering a distinct clade. Cytoform B might represent the earliest stage of speciation that can be sorted by the ECP1 gene. To test this hypothesis, we constructed a TCS haplotype network for all four Thai members. Cytoform B was distinctly separated from the main network (data not shown), indicating that it is in an early stage of reproductive isolation. However, the coalescent delimitation analyses indicate that cytoform B and a few individuals of cytoforms A, C, and K were assigned to the same GMYC entity, precluding unequivocal recognition of B as a valid species. Cytoform K forms a distinct genetic lineage based on COI, COII, and the concatenated dataset. It represents the type specimen (holotype) of *S. tani*^10^ and is supported in our analyses as a valid species. For morphoform ‘b’, all data sets—chromosomal, morphological, and molecular—indicate a distinct taxon. We, therefore recognize morphoform ‘b’ as a valid species awaiting formal taxonomic description and naming.

The assessment of species status among black flies has been complicated by the presence of cryptic species^21^,^23^. Our results further emphasize the diversification of the *S. tani* species complex in the Oriental Region. The molecular results show that *S. xuandei*, cytoform L, and morphoform ‘a’ contain possible cryptic species. However, our chromosomal analyses detected cryptic diversity only in morphoform ‘a’ (unpublished), while recognizing *S. xuandei* (unpublished) and cytoform L^9^ as distinct but single species, albeit with *S. xuandei* based on a sample of only nine larvae. A morphological re-examination of these possible cryptic taxa might help assess their potential species status.

Of the 13 taxonomic entities in our study, the chromosomes resolve ten, ECP1 resolves nine, the concatenated sequences six, and morphology five, with one taxon (morphoform ‘b’) supported by all four data sets (Table 3). In conclusion, the use of a DNA sequence-based approach supports chromosomal data for six entities. Moreover, our study reveals additional possible cryptic species, one in cytoform L, one in morphoform ‘a’ and two in *S. xuandei*, increasing taxonomic biodiversity in the *S. tani* complex by about 28% over what previously had been recognized.

## Methods

### Ethical approval

All experiments were performed in accordance with relevant guidelines and regulations of the University of Malaya. The research protocols were regulated and approved by the University of Malaya. No specific permits were required for this study, which did not involve endangered or protected species.

### Taxon sampling and species identification

Our study included 83 individuals representing six cytoforms (A, B, C, G, K, L), two morphoforms (‘a’ and ‘b’), and one recently described morphospecies (*S. xuandei*) in the *S. tani* complex from Thailand, Vietnam, Malaysia, and Taiwan (Table 1). Larval black flies were collected by hand into 1:3 acetic ethanol, and pupae and adults were fixed in 80% ethanol. Specimens were identified morphologically^12^,^27^ and then chromosomally^9^,^10^,^11^. For cytoforms A, B, C, and G, the posterior portion of the body was used for chromosomal identification and the anterior portion was used for DNA extraction. Representative specimens of each taxon are deposited in the Institute of Biological Sciences, Faculty of Science, University of Malaya (Kuala Lumpur, Malaysia) and the Clemson University Arthropod Collection (Clemson, SC, USA).

### DNA extraction, amplification, and sequencing

DNA was extracted from each specimen, using the i-genomic CTB DNA Extraction Mini Kit (iNtRON Biotechnology Inc., Seongnam, South Korea). Larvae from Thailand (cytoforms A, B, C, and G) and Vietnam (*S. xuandei*, morphoforms ‘a’ and ‘b’) were analyzed; adults from Malaysia (cytoform K, i.e., the type of *S. tani*) and Taiwan (cytoform L) were analyzed from populations previously studied chromosomally^9^,^10^,^19^. The DNA amplifications by polymerase chain reaction were conducted using an Applied Biosystems Veriti 96-Well Thermal Cycler (Applied Biosystems, Inc., Foster City, CA, USA). A preliminary assessment was performed to screen for genetic divergence, targeting the mitochondria-encoded COI, COII, 12S rRNA, and 16S rRNA, and the nuclear-encoded 28S rRNA and ECP1. However, 12S rRNA, 16S rRNA, and 28S rRNA were less variable in resolving interspecific relationships particularly in differentiating the Thai and Vietnamese members. The COI, COII, and ECP1 genes, therefore, were used as the genetic markers in this study.

Partial sequences of COI and COII were amplified using primer sets from Low *et al*.^19^ and Simon *et al*.^28^, respectively. Reaction conditions for both genes are detailed elsewhere by Low *et al*.^19^. For the ECP1 gene, an approximately 700-bp fragment was amplified using our newly designed primers: BECP1_F (5′-TGC CCT CAA ATA TCG TCA CA-3′) and BECP1_R (5′-GGC CTT CTT CAA TGT CCA AA-3′). The cycling parameters were 2 min at 94 °C, 45 s at 50 °C, and 45 s at 72 °C, followed by 36 cycles of denaturation at 94 °C for 30 s, annealing at 50 °C for 45 s, extension at 72 °C for 45 s, and a final extension at 72 °C for 4 min. The PCR products were sequenced in both directions using BIG DYE Terminator v3.1 by an ABI 3730XL Genetic Analyzer (Applied Biosystems Inc., Foster City, CA, USA). DNA sequences generated in this study are accessible from the National Center for Biotechnology Information GenBank under accession numbers KT323984-KT324057 for COI, KT324058-KT324131 for COII, and KT324141-KT324223 for ECP1.

### DNA sequence alignment

Sequences were assembled and edited using ChromasPro 1.7.6 (Technelysium Pty Ltd., Australia). All sequences were preliminarily aligned using CLUSTAL X^29^ and edited using BioEdit 7.0.9.0^30^.

### Genetic distances

Uncorrected (p) pairwise genetic distances among species were estimated using PAUP 4.0B10^31^.

### Phylogenetic analyses

To examine whether each COI, COII, and ECP1 dataset could be concatenated into a single dataset, a partition homogeneity test implemented in PAUP 4.0b10 was performed. Each separate gene region shared the same genetic information; hence, data were concatenated for further analyses.

The aligned sequences of single genes and the concatenated dataset were subjected to Bayesian inference (BI) analysis using four chains of Markov chain Monte Carlo (MCMC) implemented in MrBayes 3.1.2^32^. Four million MCMC generations were run, with convergence diagnostics calculated every 1000th generation to monitor the stabilization of log likelihood scores. Trees in each chain were sampled every 100th generation. Maximum likelihood (ML) analysis was performed with a GTR substitution model, using PhyML 3.0^33^. The branch support was evaluated using the SH-like approximate Likelihood Ratio Test (aLRT) with 1000 bootstrap replicates. Neighbour-joining (NJ) and maximum parsimony (MP) analyses were performed using PAUP 4.0b10. The NJ bootstrap values were estimated using 1000 replicates with Kimura’s two-parameter model of substitution (K2P distance). The MP tree was constructed using the heuristic search option, 100 random sequence additions, tree bisection reconnection (TBR) branch swapping, and unordered and unweighted characters. The MP Bootstrap values were computed with 1000 resamplings. *Simulium sofiani* Takaoka & Hashim and *S. tuberosum* Lundström were used as outgroups.

Substitution saturation was accessed for protein-coding DNA sequences, using DAMBE^34^,^35^. Analysis showed that all three codon positions were unsaturated (Iss values < Iss.c values). To measure the level of homoplasy, the consistency index (CI) was calculated using PAUP 4.0b10. Neither the single locus nor concatenated datasets showed any sign of pervasive homoplasy (CI > 0.5).

### Coalescent delimitation

Species delimitation among members of the *S. tani* complex was investigated using a Generalised Mixed Yule Coalescent (GMYC) model. The ultrametric tree was generated from the representative haplotypes in BEAST 1.8.2^36^ using a relaxed lognormal clock, coalescent (constant size) prior and GTR + I + G model of DNA substitution. The analysis was run for 20 million generations, with a sampling frequency of every 100 generation. The output tree was analyzed in TreeAnnotator 1.8.2 with a 10% burn-in. The data were analyzed using a single threshold model in the software package SPLITS^37^ available in R 3.2.1.

## Additional Information

**How to cite this article**: Low, V. L. *et al.* Delineating taxonomic boundaries in the largest species complex of black flies (Simuliidae) in the Oriental Region. *Sci. Rep.* **6**, 20346; doi: 10.1038/srep20346 (2016).

## Supplementary Material

Supplementary Information

## Figures and Tables

**Table 1:** Collection details for members of the *Simulium tani* complex in Asia, with GenBank accession numbers for COI, COII, and ECP1 genes.

Taxa	Locality (Code)	n	Latitude/Longitude	Collection date	GenBank Accession Number		
					COI	COII	ECP1
Cytoform A	Mork Fah Waterfall, Chiang Mai Province, Thailand (THAIA)	5	19°06′47″ 98°45′14″	17 Jan 2015	KT323984-KT323988	KT324058-KT324062	KT324141-KT324145
	Ban Lan Sang, KM16, Tak Province, Thailand (THAIAA)	6	16°48′34″ 98°59′14″	19 Feb 2014	KT323989-KT323994	KT324063-KT324068	KT324146-KT324151
Cytoform B	Klong Na Rai Waterfall, Chantaburi Province, Thailand (THAIB)	7	12°34′58″ 102°10′28″	05 Mar 2014	KT323995-KT324001	KT324069-KT324075	KT324152-KT324158
Cytoform C	Khao Lod Cave, Kanchanaburi Province, Thailand (THAIC)	7	14°40′01″ 99°19′06″	22 Feb 2012	KT324002-KT324008	KT324076-KT324082	KT324159-KT324165
Cytoform G	Ban Pang Pak, Mae Hong Son, Province, Thailand (THAIG)	7	19°26′14″ 98°21′16″	17 Jan 2015	KT324009-KT324015	KT324083-KT324089	KT324166-KT324172
Cytoform K	Cameron Highlands, Pahang Province, Malaysia (MALAYCC)	5	04°22′13″ 101°21′31″	Aug and Nov 2012	KJ636875-KJ636878*	KT324132-KT324136	KT324215-KT324219
	Cameron Highlands, Pahang Province, Malaysia (MALAYE)	4	04°28′44″ 101°22′59″	May and Aug 2012	KJ636910-KJ636913*	KT324137-KT324140	KT324220-KT324223
*S. xuandei*	Dalat, Lam Dong Province, Vietnam (VIETD)	12	12°10′56″ 108°40′48″	24 Apr 2014	KT324036- KT324047	KT324110-KT324121	KT324193-KT324204
Morphoform ‘a’	Luoi, Thua Thien Hue Province Vietnam (VIETCV)	10	16°18′16″ 107°12′48″	22 Feb 2014	KT324016-KT324025	KT324090-KT324099	KT324173-KT324182
Morphoform ‘b’	Suoi Vang Natural Forest, Lam Dong Province,Vietnam (VIETV)	10	11°59′26″ 108°22′06″	22 Apr 2014	KT324026-KT324035	KT324100-KT324109	KT324183-KT324192
Cytoform L	Waiping Village, Hsinchu County, Taiwan (TAIWT)	10	24°39′44″ 121°04′24″	3 Dec 2008	KT324048-KT324057	KT324122-KT324131	KT324205-KT324214

^*^Sequences were obtained from a previous study^19^

**Table 2:** Ranges of intraspecific and interspecific genetic distances (uncorrected *p*, expressed as percentages) among members of the *Simulium tani* complex based on COI+COII+ECP1 dataset.

	1	2	3	4	5	6	7	8	9
1. Cytoform A	0.23–1.72								
2. Cytoform B	0.00–1.95	0.74–1.21							
3. Cytoform C	0.42–1.39	0.42–1.63	0.00–1.16						
4. Cytoform G	0.46–1.77	1.07–2.00	0.51–1.49	0.70–1.67					
5. Cytoform K	1.49–2.19	1.44–2.37	1.44–1.86	1.49–2.05	0.33–1.21				
6. *S. xuandei*	2.46–3.44	2.74–3.49	2.46–3.16	2.51–3.30	2.70–3.44	0.23–1.21			
7. Morphoform ‘a’	2.60– 3.49	2.84–3.67	2.56–3.21	2.60–3.35	2.88–3.58	0.79–1.72	0.42–1.63		
8. Morphoform ‘b’	2.84–3.63	3.16–3.81	2.79–3.35	2.88–3.49	2.93–3.58	0.84–1.81	1.26–2.14	0.00–0.93	
9. Cytoform L	5.58–6.69	5.81–6.79	5.63–6.42	5.72–6.56	5.53–6.42	5.30–6.28	5.63–6.56	5.72–6.60	0.14–1.07

**Table 3:** Comparative summary of the ability of chromosomes, morphology, concatenated sequences (COI+COII+ECP1), and ECP1 sequence to distinguish taxonomic entities of the *Simulium tani* complex on mainland Southeast Asia and Taiwan.

	Chromosomes	Morphology	COI + COII + ECP1	ECP1
A	+	−	−	−
B	+	−	−	+
C	+	−	−	−
G	+	−	−	−
K	+	+	+	−
L	+	+	+	+
cryptic species	−	−	−	+
‘a’	+	+	+	+
cryptic species	+	−	−	+
‘b’	+	+	+	+
*S. xuandei*	+	+	+	+
cryptic species 1	−	−	+	+
cryptic species 2	−	−	−	+

+ = distinguishable from all other taxa; − = indistinguishable.
