# Supplementary material for: Delineating taxonomic boundaries in the largest species complex of black flies (Simuliidae) in the Oriental Region
Source: Sci Rep. 2016 Feb 3;6:20346. doi: 10.1038/srep20346 (PMC4738241; doi:10.1038/srep20346)
Supplement: Supplementary Information [file srep20346-s1.doc]

Delineating taxonomic boundaries in the largest species complex of black flies (Simuliidae) in the Oriental Region

Van Lun Low^1^, Hiroyuki Takaoka^1^, Pairot Pramual^2^, Peter H Adler^3^, Zubaidah Ya’cob^1^, Yao-Te Huang^4^, Xuan Da Pham^5^, Rosli Ramli^1^, Chee Dhang Chen^1^, Anukhcha Wannaket^1^ & Mohd Sofian-Azirun^1^

^1^Institute of Biological Sciences, Faculty of Science, University of Malaya, Kuala Lumpur, Malaysia, ^2^Department of Biology, Faculty of Science, Mahasarakham University, Maha Sarakham, Thailand, ^3^Department of Agricultural and Environmental Sciences, Clemson University, Clemson, SC, USA, ^4^Fuji Environmental Service, Mitsuwa, Kawaguchi City, Saitama, Japan and ^5^National Institute of Food Control, Ministry of Health, Hanoi, Vietnam.

Correspondence and requests for materials should be addressed to V.L.L (lucaslow24@gmail.com)

**Supplementary Table 1 |** Ranges of intraspecific and interspecific genetic distances (uncorrected p, expressed as percentages) among members of the *Simulium tani* complex based on COI gene

|  | **1** | **2** | **3** | **4** | **5** | **6** | **7** | **8** | **9** |
| --- | --- | --- | --- | --- | --- | --- | --- | --- | --- |
| **1. Cytoform A** | 0.24-2.41 |  |  |  |  |  |  |  |  |
| **2. Cytoform B** | 1.20-2.65 | 0.00-1.44 |  |  |  |  |  |  |  |
| **3. Cytoform C** | 0.00-2.29 | 0.00-2.29 | 0.00-2.17 |  |  |  |  |  |  |
| **4. Cytoform G** | 0.24-2.41 | 1.20-2.41 | 0.72-2.29 | 0.48-2.41 |  |  |  |  |  |
| **5. Cytoform K** | 2.05-3.37 | 1.68-3.37 | 2.29-3.25 | 1.93-3.37 | 0.12-1.68 |  |  |  |  |
| **6. *S. xuandei*** | 3.37-4.57 | 3.37-4.45 | 3.49-4.45 | 3.25-4.33 | 3.25-4.69 | 0.00-1.56 |  |  |  |
| **7. Morphoform ‘a’** | 3.37-4.57 | 3.49-4.69 | 3.49-4.57 | 3.37-4.45 | 3.37-4.81 | 0.24-1.93 | 0.36-2.05 |  |  |
| **8. Morphoform ‘b’** | 3.37-4.45 | 3.13-4.57 | 3.37-4.45 | 3.25-4.21 | 2.89-4.57 | 0.12-1.81 | 0.36-2.17 | 0.00-1.81 |  |
| **9. Cytoform L** | 7.58-8.66 | 7.34-8.54 | 7.58-8.30 | 7.58-8.54 | 6.86-7.94 | 7.46-8.42 | 7.73-8.54 | 7.46-8.42 | 0.00-0.72 |

**Supplementary Table 2 |** Ranges of intraspecific and interspecific genetic distances (uncorrected *p*, expressed as percentages) among members of the *Simulium tani* complex based on COII gene

|  | **1** | **2** | **3** | **4** | **5** | **6** | **7** | **8** | **9** |
| --- | --- | --- | --- | --- | --- | --- | --- | --- | --- |
| **1. Cytoform A** | 0.00-1.51 |  |  |  |  |  |  |  |  |
| **2. Cytoform B** | 0.30-1.36 | 0.00-1.20 |  |  |  |  |  |  |  |
| **3. Cytoform C** | 0.00-1.05 | 0.00-0.90 | 0.00-0.30 |  |  |  |  |  |  |
| **4. Cytoform G** | 0.00-1.81 | 0.30-1.66 | 0.00-1.36 | 0.15-1.66 |  |  |  |  |  |
| **5. Cytoform K** | 0.90-1.96 | 1.05-2.11 | 1.05-1.51 | 0.75-1.96 | 0.00-0.75 |  |  |  |  |
| **6. *S. xuandei*** | 1.81-3.16 | 1.81-3.01 | 1.81-2.41 | 1.81-2.86 | 2.11-2.71 | 0.00-1.20 |  |  |  |
| **7. Morphoform ‘a’** | 1.66-3.16 | 1.81-2.86 | 1.66-2.41 | 1.66-3.16 | 1.96-3.01 | 0.15-1.51 | 0.00-1.51 |  |  |
| **8. Morphoform ‘b’** | 1.51-2.86 | 1.81-2.71 | 1.51-2.11 | 1.81-2.86 | 1.96-2.71 | 0.00-1.20 | 0.00-1.20 | 0.00-0.90 |  |
| **9. Cytoform L** | 6.02-7.38 | 6.33-7.68 | 6.33-7.08 | 6.02-7.23 | 6.48-7.68 | 6.48-7.53 | 6.33-7.83 | 6.48-7.53 | 0.00-0.75 |

**Supplementary Table 3 |**  Ranges of intraspecific and interspecific genetic distances (uncorrected *p*, expressed as percentages) among members of the *Simulium tani* complex based on ECP1 gene

|  | **1** | **2** | **3** | **4** | **5** | **6** | **7** | **8** | **9** |
| --- | --- | --- | --- | --- | --- | --- | --- | --- | --- |
| **1. Cytoform A** | 0.00-2.13 |  |  |  |  |  |  |  |  |
| **2. Cytoform B** | 1.22-2.59 | 0.00-1.68 |  |  |  |  |  |  |  |
| **3. Cytoform C** | 0.61-1.68 | 1.22-2.13 | 0.00-0.76 |  |  |  |  |  |  |
| **4. Cytoform G** | 0.46-2.13 | 0.91-2.90 | 0.46-1.52 | 0.76-1.98 |  |  |  |  |  |
| **5. Cytoform K** | 0.46-2.13 | 0.76-2.59 | 0.30-1.22 | 0.30-1.83 | 0.00-1.37 |  |  |  |  |
| **6. *S. xuandei*** | 1.37-3.66 | 2.29-4.12 | 1.68-2.74 | 1.68-3.05 | 1.83-3.05 | 0.00-1.37 |  |  |  |
| **7. Morphoform ‘a’** | 1.52-3.66 | 2.44-4.27 | 1.68-2.90 | 1.68-3.35 | 1.83-3.05 | 1.52-2.90 | 0.00-2.59 |  |  |
| **8. Morphoform ‘b’** | 3.05-4.42 | 3.96-4.57 | 3.05-3.51 | 3.05-3.81 | 2.90-3.66 | 2.29-3.05 | 2.74-3.81 | 0.00 |  |
| **9. Cytoform L** | 1.37-4.42 | 2.59-4.57 | 1.68-3.51 | 1.68-3.81 | 1.83-3.81 | 0.46-2.74 | 1.52-3.66 | 1.29-3.66 | 0.00-2.29 |


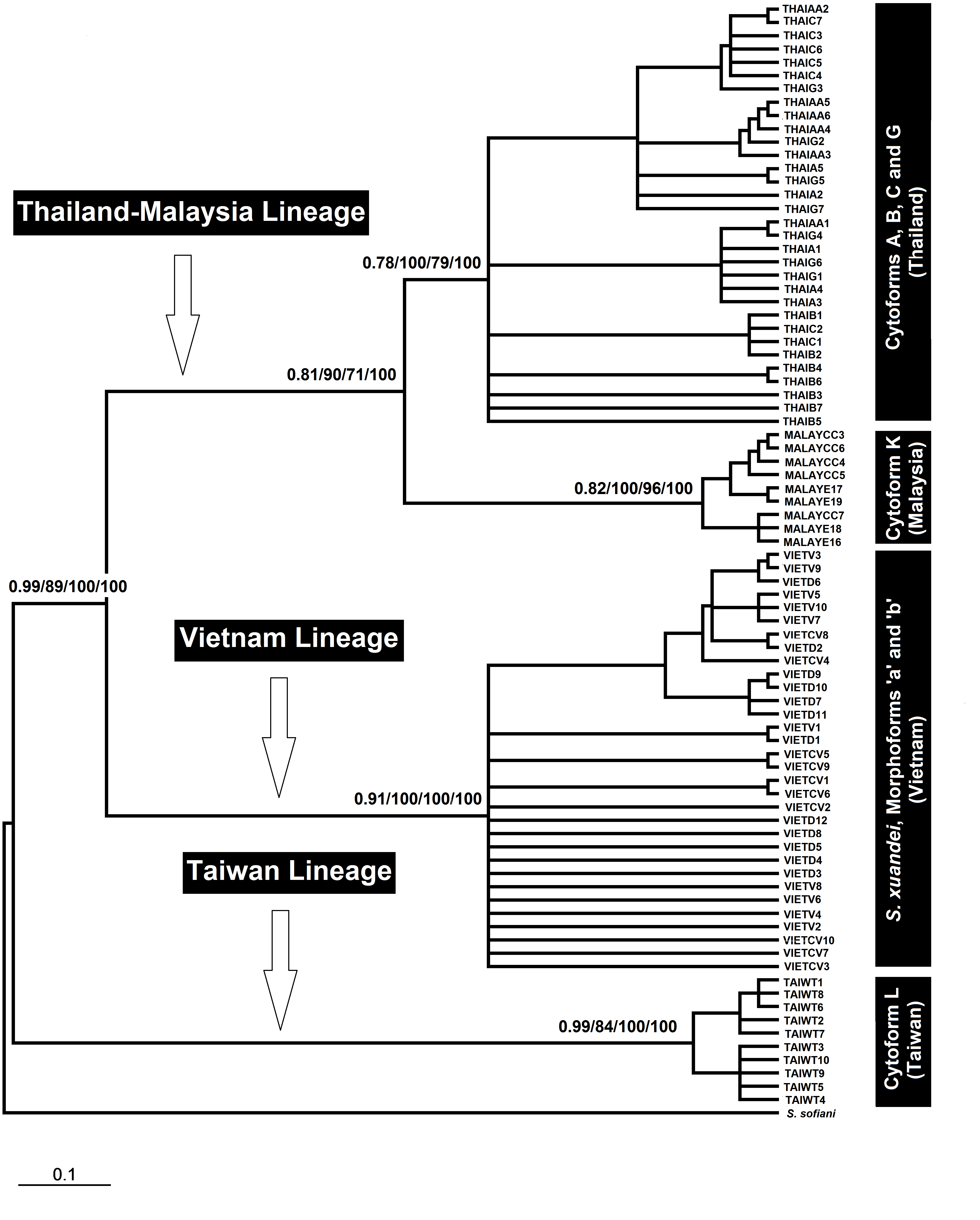


**Supplementary Figure 1 |**  Phylogenetic tree of *Simulium* taxa based on COI sequences. Posterior probability/bootstrap [Bayesian inference (BI)/maximum likelihood (ML)/neighbour-joining (NJ)/maximum parsimony (MP)] values are shown on the branches. Values less than 0.5/50 are not shown. The scale bar represents 0.1 substitutions per nucleotide position.


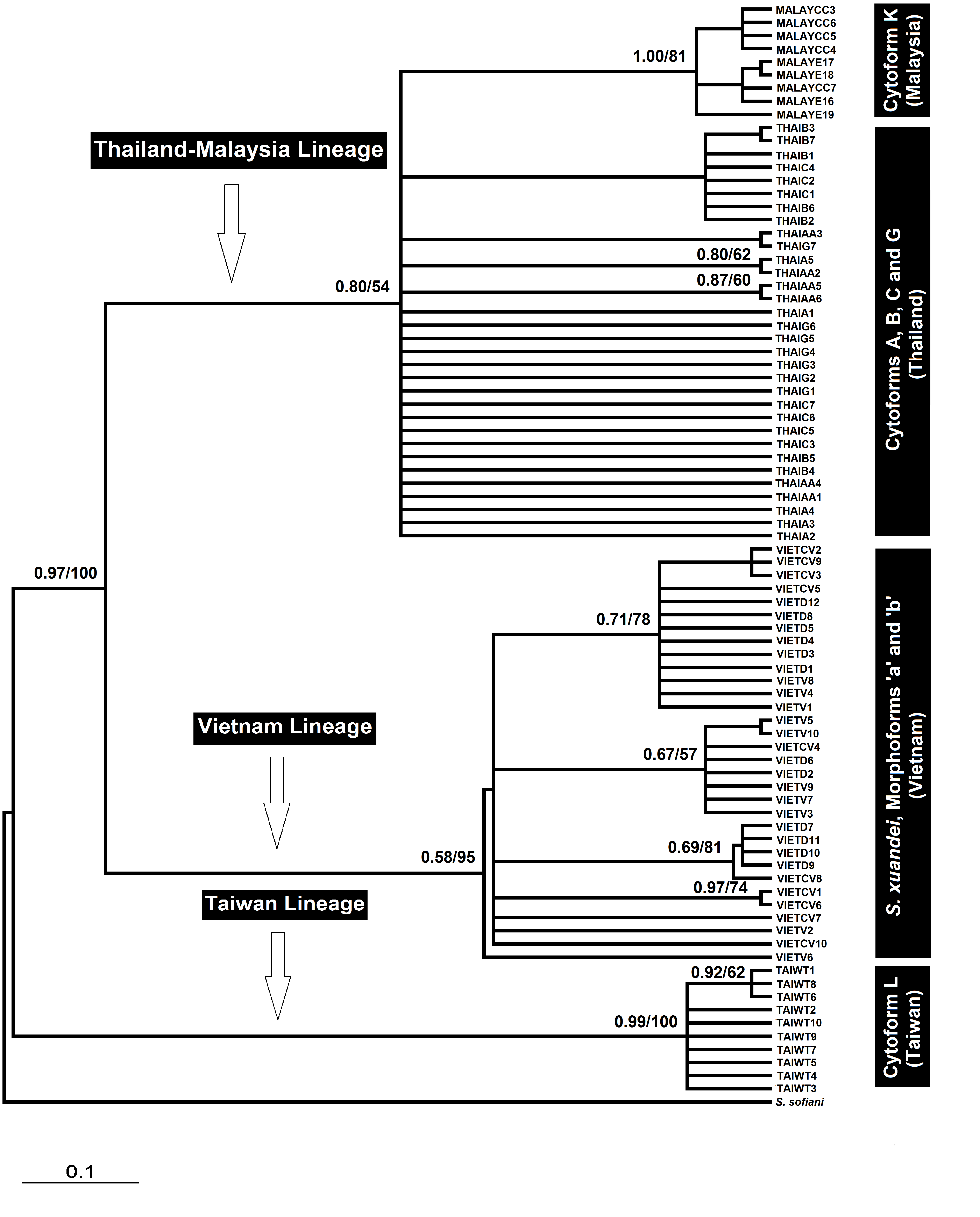


**Supplementary Figure 2 |** Phylogenetic tree of *Simulium* taxa based on COII sequences. Posterior probability/bootstrap [Bayesian inference (BI)/neighbour-joining (NJ)] values are shown on the branches. Values less than 0.5/50 are not shown. The scale bar represents 0.1 substitutions per nucleotide position.
